# Bacterial Isolates and Antibiotic Susceptibility Patterns in Sterile Body Fluids in Patients Attending a Tertiary Care Hospital: A Two-Year Retrospective Study

**DOI:** 10.7759/cureus.104772

**Published:** 2026-03-06

**Authors:** Nirmala Poddar, Adrita Das, Smaranita Sabat, Dipti Pattnaik, Kumudini Panigrahi

**Affiliations:** 1 Microbiology, Kalinga Institute of Medical Sciences, Bhubaneswar, IND; 2 Community Medicine, Institute of Medical Sciences and SUM Hospital, Bhubaneswar, IND

**Keywords:** antibiotic susceptibility, cerebrospinal fluid, mrsa, multidrug resistance, pleural fluid, sterile body fluids, synovial fluid

## Abstract

Introduction

Infections of sterile body fluids are caused by various bacterial agents and are usually associated with high morbidity and mortality. Timely microbiological culture and sensitivity testing of clinical specimens can identify the etiological agent, guide the targeted antibiotics, and reduce further complications. Knowledge of local antimicrobial susceptibility patterns is essential for selecting appropriate empirical therapy. The present study aimed to determine the profile of bacterial pathogens and their antimicrobial resistance patterns isolated from normally sterile body fluids at a tertiary care center in Odisha, India.

Methods

A retrospective laboratory-based observational study was carried out in the Department of Microbiology, Kalinga Institute of Medical Sciences, Bhubaneswar, Odisha. Culture and sensitivity records from the previous two years (January 2021 to December 2022) were retrieved and analyzed after getting approval by the Institutional Ethics Committee (KIIT/KIMS/IEC/1098/2023, date: January 1, 2023).

A total of 2307 sterile body fluid samples (CSF, pleural, ascitic, and synovial fluids) were processed using standard microbiological procedures. Isolate identification and antimicrobial susceptibility testing were carried out using the VITEK-2 system and interpreted according to Clinical and Laboratory Standards Institute (CLSI) guidelines. Data were analyzed using SPSS version 26.0 (IBM Corp., Armonk, NY).

Results

Out of 2307 samples, 291 yielded bacterial growth, giving an overall culture positivity rate of 12.6%. Pleural fluids showed the highest positivity rate (18.5%), followed by synovial fluid (16.4%). Gram-positive bacteria outnumbered (54%), with *Staphylococcus aureus* as the most common isolate (31.6%). Among Gram-negative isolates, *Klebsiella pneumoniae* (13.7%) was predominant. *Enterobacterales* showed higher susceptibility to aminoglycosides and carbapenems, with high resistance to ciprofloxacin. *Pseudomonas *spp. and *Acinetobacter *spp. exhibited limited susceptibility to the commonly used antibiotics. Multidrug-resistant (MDR), extensively drug-resistant (XDR), and pandrug-resistant (PDR) among Gram-negative bacilli (GNB) were 21.6%, 14.2%, and 3%, respectively. Methicillin-resistant *Staphylococcus aureus* (MRSA) constituted 48.9% of *Staphylococcus aureus*.

Conclusion

Sterile body fluid infections due to bacterial agents at our center were found to be 12.6% and showed a diverse bacterial profile with a substantial burden of antimicrobial resistance. The high prevalence of MDR GNB and MRSA underscores the importance of continuous local surveillance and antimicrobial stewardship to guide empirical therapy and improve patient outcomes.

## Introduction

The fluids normally present in the sterile body compartments are known as sterile body fluids. In heathy state, the sterile body fluids are free from any kind of microbes. Common types of sterile body fluids include cerebrospinal fluid (CSF), ascitic fluid, pleural fluid, and synovial fluid, which are sent to the microbiology laboratory for culture and sensitivity testing in clinically suspected infection cases [1,2].

A wide range of pathogens, such as bacteria, fungi, viruses, and parasites, can breach normally sterile body compartments, invade the sterile body fluids, causing severe infections, leading to high morbidity and mortality [1]. Hence, timely diagnosis and prompt initiation of appropriate antimicrobial therapy are necessary for combating these infections and improving clinical outcomes [1-4].

Bacterial pathogens are the commonly isolated organisms from sterile body fluids. The common Gram-negative bacterial agents of sterile body fluid infections include *Escherichia coli*, *Klebsiella* spp, *Acinetobacter* spp., *Pseudomonas* spp., and *Neisseria meningitidis*, and common Gram-positive bacteria include *Staphylococcus* spp., *Streptococcus* spp., and *Enterococcus* spp. [5,6].

The epidemiology of these pathogens varies according to the type of sterile body fluid involved. *Streptococcus pneumoniae* and *Neisseria meningitidis* are commonly associated with CSF infections, whereas infection of ascitic and peritoneal fluids is frequently (40-60% of the cases) associated with Gram-negative enteric bacteria such as *Escherichia coli* and *Klebsiella* spp. Pleural and synovial fluid infections are more often caused by Gram-positive organisms like *Staphylococcus aureus* and *Streptococcus* spp. [4,5].

Site-specific variations in the etiological agents of the body fluid infection highlight the importance of understanding the local pathogen distribution and guides the clinicians for appropriate empirical therapy [4-6].

Many times, these infections are caused by antimicrobial-resistant bacteria, which lead to reduced response to treatment, leading to longer hospital stays, higher medical costs, and increased mortality [4,7,8]. To a great extent, it is more prevalent in developing countries due to limited health care services, scarce resources, poor hygiene and sanitation, and irrational use of antibiotics [5]. Global reports indicate that the prevalence of sterile body fluid infections ranges from 7.6 % to 14.9%, with a mortality rate reaching up to 20%, and nearly 60% of the isolates are showing antimicrobial resistance [8,9]. In contrast, data from a tertiary care hospital in North India have shown a higher prevalence of about 32-50% [10]. Similarly, Tiwari et al. reported a prevalence of 28% from a tertiary care hospital in Odisha, showing a high prevalence rate of bacterial infection in sterile body fluids in this region [11].

Advancements in microbial identification and antibiotic susceptibility testing by automated methods, such as the bioMérieux VITEK 2 system, have improved the accuracy as well as rapidity of pathogen detection and provide reliable antimicrobial susceptibility results in clinical microbiology. Both identification of the clinically significant pathogen and its antimicrobial susceptibility testing (AST) reports are essential for guiding appropriate therapy.

Although several studies have been conducted on the microbiological profile of sterile body fluid infections, variations in pathogen distribution and antimicrobial resistance patterns exist across regions and over time. Therefore, continuous institution-specific surveillance is essential to understand local epidemiological trends, develop an antibiogram, and guide antimicrobial stewardship.

Hence, the present study was carried out with the aim of determining the bacterial profile and antimicrobial susceptibility patterns of clinically significant bacterial isolates in sterile body fluids at a tertiary care center in Odisha, India.

## Materials and methods

A retrospective laboratory-based observational study was conducted at the Department of Microbiology, Kalinga Institute of Medical Sciences, Bhubaneswar, Odisha, after getting IEC approval (KIIT/KIMS/IEC/1098/2023, date: January 1, 2023). Culture and sensitivity records of sterile body fluid samples from January 2021 to December 2022 were retrieved and analyzed only after IEC approval.

A total of 2307 body fluid samples were received during the two-year period. Sterile body fluids (CSF, ascitic fluid, pleural fluid, synovial fluid) received from patients of all ages and sexes, from both inpatient and outpatient departments, were included. Blood samples, samples showing mixed growth (collection/handling contamination) were regarded as contaminated samples, and delayed transported samples for more than two hours were excluded.

Procedure

The sterile body fluid specimens were received in the microbiology laboratory after proper collection and transport. They were processed immediately in the laboratory without refrigeration, using standard microbiological procedures. Direct smears were made from each specimen for Gram staining. The specimens were inoculated onto blood agar, MacConkey agar, and chocolate agar plates, and 1-3 mL of the specimen was inoculated into pediatric blood culture bottles of the BacT/ALERT system after following standard aseptic measures.

The inoculated culture media were incubated at 37°C for 24 to 48 hours in a carbon dioxide incubator; the blood culture bottles were incubated in the BacT/ALERT 3D system for a maximum of five days. From the positive culture plates and bottles, subcultures were made to obtain the isolates. Isolate identification and antibiotic sensitivity testing were done by the automated VITEK 2 compact system. Interpretation of antibiotic susceptibility was done as per CLSI 2022 guidelines [12].

The antibiotics tested for Gram-positive cocci (GPC) included benzylpenicillin, gentamicin, ciprofloxacin, clindamycin, erythromycin, cotrimoxazole, linezolid, daptomycin, tigecycline, teicoplanin, and vancomycin. The antibiotics tested for Gram-negative bacilli (GNB) isolates were ampicillin, amoxicillin/clavulanic acid, amikacin, gentamicin, ceftriaxone, ceftazidime, piperacillin/tazobactam, cefepime, ciprofloxacin, cotrimoxazole, meropenem, imipenem, and tigecycline.

Data were collected from registers regarding patients’ demography (age, sex), and culture and sensitivity data (organisms, AST profile) were retrieved from laboratory registers and entered into Microsoft Excel (Microsoft® Corp., Redmond, WA). Statistical analysis was carried out using SPSS version 26.0 (IBM Corp., Armonk, NY). Categorical data were presented as frequencies and percentages. The association between the type of body fluid and culture positivity was evaluated using the chi-square test. A p-value <0.05 was considered statistically significant. Confidence intervals were not calculated, as the study was descriptive in nature.

## Results

The present study included a total of 2307 sterile body fluid samples received in the microbiology laboratory during the study period for culture and sensitivity testing. The samples comprised CSF (n = 1121, 48.6%), pleural fluid (n = 518, 22.4%), ascitic fluid (n = 593, 25.7%), and synovial fluid (n = 75, 3.2%).

Bacterial growth was found in 291 samples, yielding an overall culture positivity rate of 12.61%. The majority of positive cases were males, 146/291 (67%), with a mean age of 48 ± 21 years.

Table 1 shows the distribution of culture positivity among different sterile body fluid samples (n = 2307). Culture positivity rate was highest in pleural fluid (96/518, 18.5%) and lowest in CSF (96/1121, 8.5%). A statistically significant association was found between the types of sterile body fluid and culture positivity. 

**Table 1:** Distribution of culture positivity among different sterile body fluid samples (n = 2307) Data were expressed as n (%). The chi-square test was used, and chi-square values were given along with the p-values. A p-value <0.05 was considered statistically significant.

Sample type	Total samples	Growth n (%)	No growth n (%)	Statistical test used	Test statistics	p-value
Cerebrospinal fluid	1121	96 (8.50%)	1025 (91.50%)	Chi-square test	37.28	<0.001
Pleural fluid	518	96 (18.50%)	422 (81.50%)	Chi-square test	18.94	<0.001
Synovial fluid	75	12 (16.00%)	63 (84.00%)	Chi-square test	7.18	<0.001
Ascitic fluid	593	87 (14.67%)	506 (85.33%)	Chi-square test	23.23	<0.001
Total	2307	291 (12.61%)	2016 (87.39%)	Chi-square test	34.73	<0.001

Table 2 shows the frequency of distribution of bacterial isolates by type of sterile body fluids. Among the 291 bacterial isolates, Gram-positive bacteria predominated (157/291, 53.95%) with *Staphylococcus aureus* as the most frequently isolated organism (92/291, 31.6). Among Gram-negative isolates, *Klebsiella pneumoniae* (40/291; 13.7%) was the predominant isolate, followed by *Escherichia coli* (27/291; 9.3%). *Staphylococcus aureus* was the most frequent pathogen in both CSF and pleural fluid, while *Klebsiella pneumoniae* and *Escherichia coli* predominated in ascitic fluid.

**Table 2:** Frequency of distribution of bacterial isolates by type of sterile body fluids (n = 291) Data are expressed as n (%). Percentages are calculated column-wise based on total isolates in each fluid type. CSF, cerebrospinal fluid; CONS, coagulase-negative *Staphylococcus aureus*

Bacterial isolates		Cerebrospinal fluid (n = 96) n (%)	Pleural fluid (n = 96) n (%)	Synovial fluid (n = 12) n (%)	Ascitic fluid (n = 87) n (%)	Total (n = 291) n (%)
Gram-positive bacterial isolate (n = 157),	Streptococcus pneumoniae	2(2.9%)	0	0	1 (1.1%)	3 (1.0%)
	Staphylococcus aureus	36 (37.5%)	32 (33.3%)	4 (33.3%)	20 (23.0%)	92 (31.6%)
	CONS	22 (23.0%)	8 (8.2%)	2 (16.6%)	11(12.5%)	43 (14.7%)
	*Enterococcus* spp.	8 (8.3%)	2 (2.1%)	0	5 (5.8%)	15 (5.1%)
	*Streptococcus* spp.	2 (2.08%)	0	1 (8.3%)	0	3 (1.03%)
	Granulicatella adiacens	1 (1.04%)	0	0	0	1 (0.3%)
Gram-negative bacterial isolate (n = 134)	Klebsiella pneumoniae	8 (8.3%)	23 (23.9%)	0	9 (10.3%)	40 (13.8%)
	Escherichia coli	0	9 (9.4%)	0	18 (20.6%)	27 (9.3%)
	*Pseudomonas* spp.	3 (3.1%)	4 (4.2%)	3 (25.0%)	9 (10.3%)	19 (6.5%)
	*Citrobacter* spp.	0	1 (1.0%)	0	0	1 (0.3%)
	*Acinetobacter* spp.	12 (12.5%)	4 (4.2%)	0	8 (9.2%)	24 (8.2%)
	*Enterobacter* spp.	1 (1.0%)	4 (4.2%)	1 (8.3%)	0	6 (2.1%)
	Burkholderia cepacia	0	3 (3.1%)	1 (8.3%)	2 (2.3%)	6 (2.1%)
	Stenotrophomonas maltophilia	1 (1.0%)	2 (2.1%)	0	0	3 (1.0%)
	*Sphingomons* spp.	0	3 (3.1%)	0	3 (3.4%)	6 (2.1%)
	*Providencia* spp.	0	1 (1.0%)	0	0	1 (0.3%)
	*Salmonella* spp.	0	0	0	1 (1.1%)	1 (0.3%)

**Figure 1:**
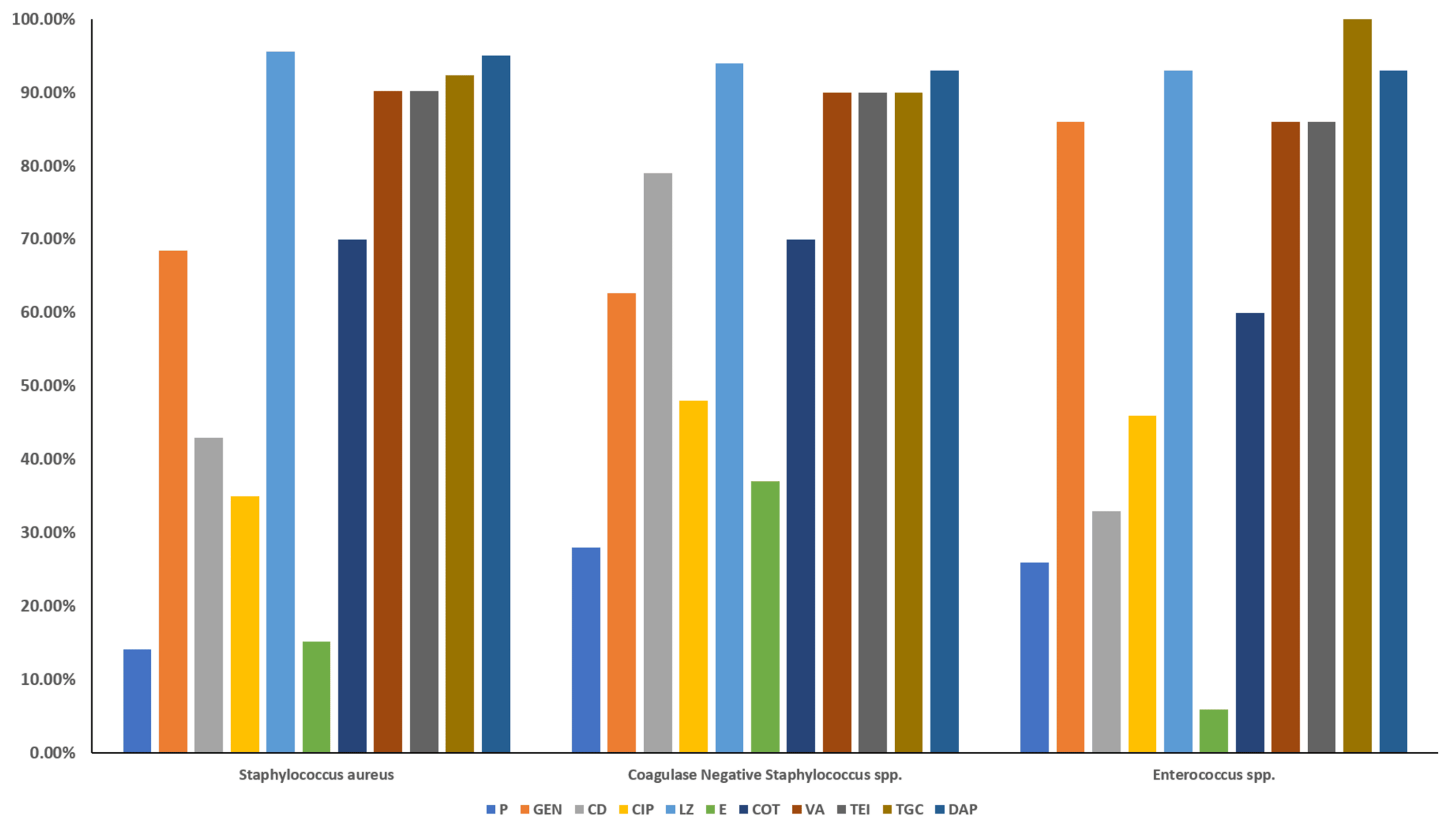
Antibiotic susceptibility pattern of Gram-positive bacterial isolates from various sterile body fluids Bar diagram showing the percentage sensitivity of Gram-positive isolates to the tested antibiotics. Data are expressed as a percentage of isolates sensitive. Antibiotics tested are the following: CD, clindamycin; CIP, ciprofloxacin; COT, cotrimoxazole; DAP, daptomycin; E, erythromycin; G, gentamicin; LZ, linezolid; P, penicillin; TEI, teicoplanin; TGC, tigecycline; VA, vancomycin

Figure 1 illustrates the pattern of antibiotic susceptibility in Gram-positive bacterial isolates. Among the Gram-positive isolates, *Staphylococcus* spp. showed the highest sensitivity to linezolid (95.65%) and daptomycin (95%), followed by tigecycline (92%), vancomycin, and teicoplanin (90.21% each). *Enterococcus* spp. were highly sensitive to tigecycline (100%), followed by daptomycin and linezolid (93% each) and vancomycin, teicoplanin, and high-level gentamicin (86% each). *Streptococcus* spp. were 100% sensitive to benzylpenicillin, daptomycin, gentamicin, linezolid, and vancomycin.

**Figure 2:**
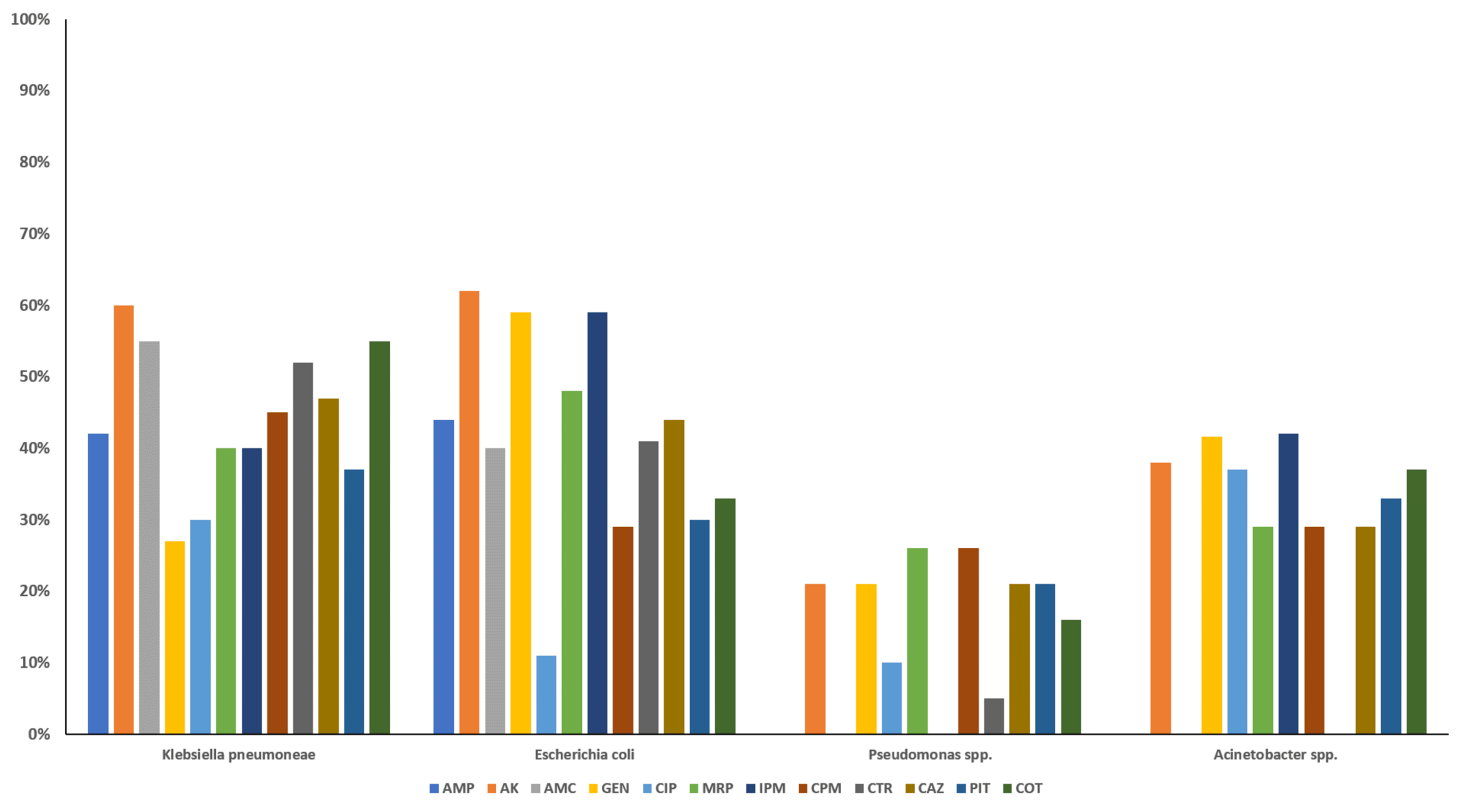
Antibiotic susceptibility pattern of Gram-negative bacterial isolates from various sterile body fluids Bar diagram showing the percentage sensitivity of Gram-negative bacilli to the tested antibiotics. Data are expressed as a percentage of isolates sensitive. The antibiotics are the following: AK, amikacin; AMC, amoxiclav; AMP, ampicillin; CAZ, ceftazidime; CIP, ciprofloxacin; COT, cotrimoxazole; CPM, cefepime; CTR, ceftriaxone; GEN, gentamicin; IPM, imipenem; MRP, meropenem

Figure 2 illustrates the antibiotic susceptibility pattern of Gram-negative bacterial isolates. Gram-negative *Enterobacterales* (*Escherichia coli* and *Klebsiella pneumoniae*) showed moderate sensitivity to amikacin (41%-63%) and carbapenems (40%-60%). Ciprofloxacin resistance was high (90%). *Pseudomonas* spp. were also moderately sensitive to amikacin, followed by carbapenems, with low and variable susceptibility to piperacillin-tazobactam (21-37%) and ciprofloxacin (10-11%). *Acinetobacter* spp. demonstrated 41% sensitivity to aminoglycosides and imipenem, and slightly lower susceptibility to ciprofloxacin (38%) and cotrimoxazole (37.5%).

**Figure 3:**
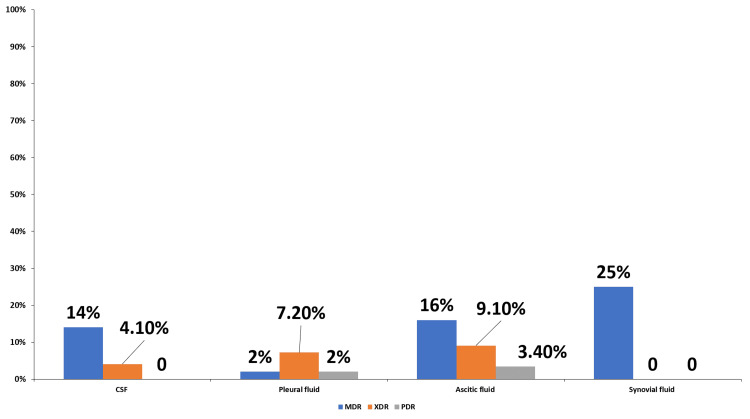
Distribution of MDR, XDR, and PDR among Gram-negative bacilli isolates The bar chart shows the percentage of MDR, XDR, and PDR strains among Gram-negative bacilli isolated from various sterile body fluids. Values above bars indicate the percentage of isolates in each resistance category. CSF, cerebrospinal fluid; MDR, multidrug-resistant; PDR, pandrug-resistant; XDR, extensively drug-resistant

Figure 3 shows that among GNB (n = 134), the prevalence of multidrug-resistant (MDR), extensively drug-resistant (XDR), and pandrug-resistant (PDR) strains was 29/134 (21.6%), 19/134 (14.2%), and 4/134 (3%), respectively. MDR strains were most frequent in synovial fluid (3/5, 60%) and ascitic fluid (14/50, 28%). Ascitic fluid also accounted for the majority of XDR (8/50, 16%) and PDR (3/50, 16%) isolates.

**Figure 4:**
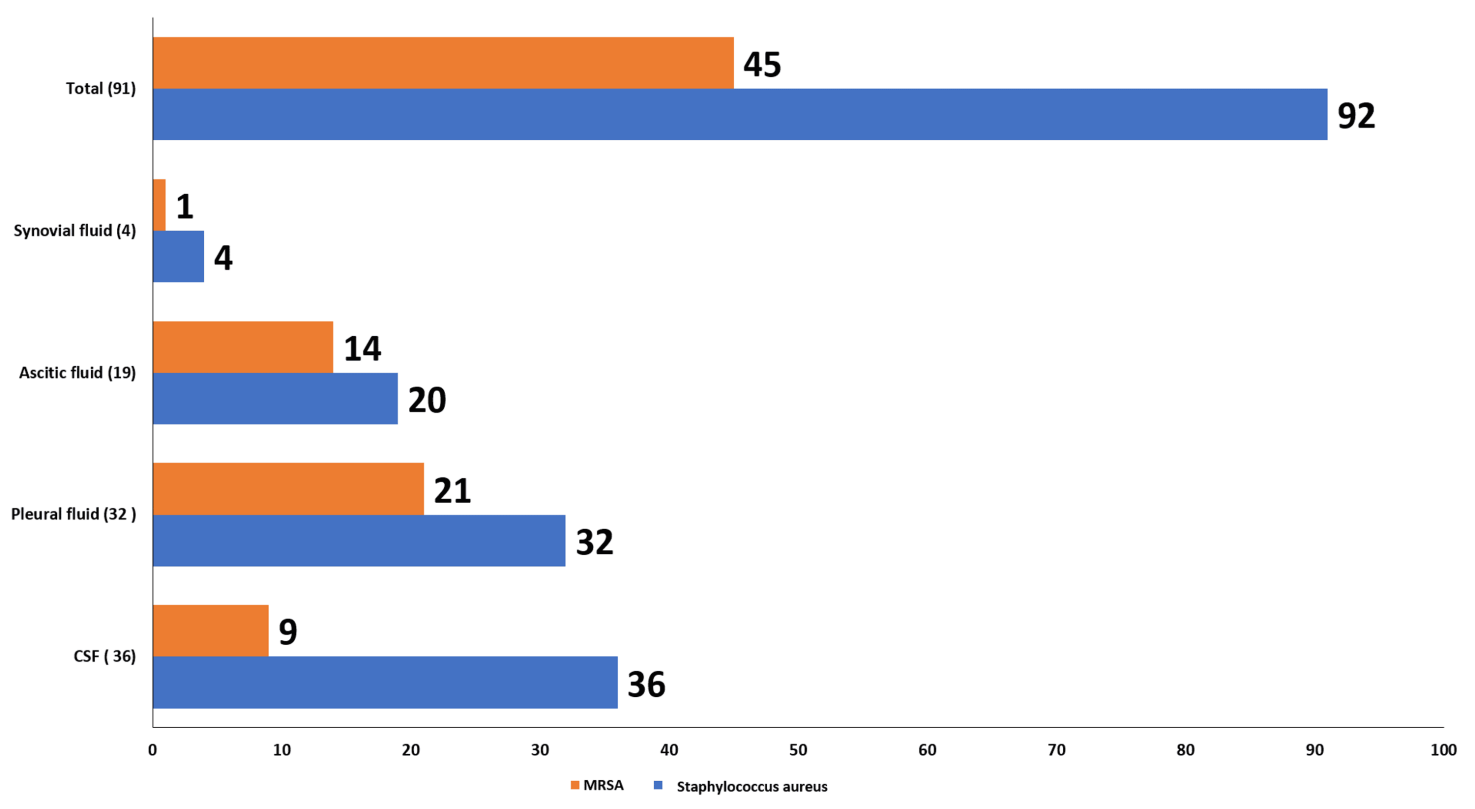
Distribution of MRSA among Staphylococcus aureus isolates from different sterile body fluids Bar diagram showing the number (N) of MRSA isolates out of the total *Staphylococcus aureus* isolates in each sterile body fluid. Values above the bars indicate MRSA count/total *Staphylococcus aureus isolates* for each specimen type. CSF, cerebrospinal fluid; MRSA, methicillin-resistant *Staphylococcus aureus*

Figure 4 shows that among *Staphylococcus aureus* isolates (n = 92), the prevalence of MRSA strains was 45/92 (48.9%). The isolation rate of MRSA was highest in ascitic fluid (14/19), followed by pleural fluid specimens (21/32).

## Discussion

Infection of sterile body fluids can result in severe morbidity and mortality. The microorganisms responsible for infections of sterile body fluids, as well as their sensitivity patterns, vary with the types of fluid infected. Additionally, the rising prevalence of multidrug-resistant strains is creating a significant healthcare concern [8]. Thus, there is a need for local surveillance data on appropriate identification and antibiogram profiling of these microorganisms to guide empirical therapy for patients.

The present study observed an overall culture positivity rate of 12.61% (291/2307) in sterile body fluids. Findings of our study were comparable to the findings reported by Flurin et al. [13] and Cox et al. [14]. Singh et al. [9] documented the rate as 9.69%, and Mohanty et al. [15] reported a positivity rate of 15.8%. A recent article by Chen et al. [16] performed metagenomic next-generation sequencing of body fluids and found similar results. However, a higher culture positivity rate (28%) was reported by Tiwari et al. [11] from Odisha. Studies done by Sharma and Anuradha [1] and Sujatha et al. [17] reported a high rate of bacterial growth, 30% and 31%, respectively. These variabilities in growth patterns may be due to regional variations in patient characteristics, prior antibiotic use, and laboratory methods used in diagnosis. In the present study, the highest culture positivity rate was observed in pleural fluid (18.5%), followed by synovial fluid 16.4%. A similar pattern of distribution was found in other Indian studies [1,9].

The majority of isolated bacteria were of the Gram-positive category (54%) with *Staphylococcus aureus* as the leading pathogen, as shown in Table 2. Among Gram-negative bacteria, *Klebsiella pneumoniae* was the most commonly isolated, followed by *Escherichia coli*, as shown in Table 2. In contrast, studies by Sharma and Anuradha [1], Sujatha et al. [17], and Sheikhbahaei et al. [18] reported *Escherichia coli* followed by *Staphylococcus aureus* to be the predominant organism. However, a study from Odisha by Tiwari et al. [11] found GNB (69.8%) as the predominant infecting agent, with *Escherichia coli* followed by *Klebsiella pneumoniae*. The above observations suggest that the causative organism of sterile body fluid infections varies according to different places as well as regions.

In CSF, *Staphylococcus aureus* was the commonly isolated organism, followed by *Acinetobacter baumannii*, whereas studies by Rouf et al. [4] and by Feng et al. [19] reported *Acinetobacter* spp. as the predominant organism. In pleural fluid specimens of our study, *Staphylococcus aureus* was the common isolate, followed by *Klebsiella pneumoniae*, which is consistent with findings of other studies [19]. Whereas studies by Gopalakrishnan et al. found *Acinetobacter* spp. and *Escherichia coli* as the most common organisms [20]. Sujatha et al. [17] and Ren et al. [21] reported *Klebsiella* spp. and *Escherichia coli* as the common isolates.

In ascitic fluid, *Staphylococcus aureus* was the common isolate, followed by *Escherichia coli*, as shown in Table 2. A similar finding was reported by Vishalakshi et al. [22]. However, various studies from India have reported *Escherichia coli* to be the predominant organism causing ascitic fluid infection [4,18,21]. In case of synovial fluid, *Staphylococcus aureus* was the most common isolate (4, 33.3%), followed by *Pseudomonas aeruginosa*. Our results of synovial fluid culture are consistent with earlier studies by Rouf et al. [4] and Vishalakshi et al. [22]. This may be due to the skin and mucous membrane colonizing property shown by the pathogen and its diverse virulence factors, resulting in invasion and infection of sterile body sites.

Our findings suggest that Gram-negative *Enterobacterales* were mostly sensitive to aminoglycosides (41-63%), followed by carbapenems (40-60%), and were markedly resistant to ciprofloxacin (90%). Similar observations are reported by Feng et al. [19] and Barai et al. [23], who also mentioned amikacin as the most effective, and cephalosporins and fluoroquinolones are highly resistant (>80) antibiotics for *Escherichia coli* isolates.

Among non-fermenters, *Pseudomonas* spp. showed the highest sensitivity to amikacin, followed by carbapenems, and were less sensitive to piperacillin-tazobactam and ceftazidime (21% each) and to fluoroquinolones (10-11%). In contrast, various previous studies reported a higher susceptibility of *Pseudomonas* spp. to piperacillin-tazobactam and carbapenems [1]. *Acinetobacter* spp. were mostly sensitive to aminoglycosides (41%) and imipenem (41%).

*Staphylococcus aureus* showed high susceptibility to linezolid, daptomycin, tigecycline, and glycopeptides. *Enterococcus* spp. were 100% sensitive to tigecycline, followed by daptomycin, linezolid, and glycopeptides. Isolated *Streptococcus* spp. were 100% sensitive to all the tested antibiotics. These observed high resistance levels may be attributed to the inappropriate and irrational use of commonly prescribed antibiotics. Prevalence of MDR, XDR, and PDR Gram-negative isolates 10%, 6.5%, and 1.3%, respectively, with the highest burden in synovial fluids, followed by ascitic fluid. MRSA accounted for 48.9% of *Staphylococcus aureus* isolates, with higher prevalence in ascitic fluid. The above findings may be due to regional differences in antimicrobial resistance patterns and antibiotic exposure of the population.

Findings of our study indicate a rising prevalence of MDR, XDR, extended-spectrum β-lactamases (ESBL), and MRSA strains over recent years. There has been a global surge in the emergence of drug-resistant organisms in recent years. Several previous studies have reported that MRSA is increasing as a significant cause of community-acquired infections with an overall prevalence of 30% to 90% according to the type of infection [9,15].

Limitations

This study has several limitations. Being a retrospective, single-center laboratory-based study, clinical correlation could not be done. A multicentric prospective study is required for a better understanding of the causative agent. Molecular characterization of the resistance gene is needed for exploring the mechanisms of resistance.

## Conclusions

This study identified an infection rate of 12.6% in normally sterile body fluids. *Staphylococcus aureus*, *Klebsiella* spp., and *Acinetobacter baumannii* were the most frequently isolated organisms. An increasing trend of antimicrobial resistance was observed among the isolated pathogens to the commonly used antibiotics. The high prevalence of infections due to MDR GNB and MRSA strains highlights the need for continuous surveillance of those organisms. Implementation of a local antibiogram and strict antimicrobial stewardship program is essential for effective management of body fluid infections.
